# Diterpenoid Compounds Isolated from *Chloranthus oldhamii* Solms Exert Anti-Inflammatory Effects by Inhibiting the IKK/NF-κB Pathway

**DOI:** 10.3390/molecules26216540

**Published:** 2021-10-29

**Authors:** Lin-Chieh Chiu, Jir-You Wang, Chao-Hsiung Lin, Chung-Hua Hsu, Lie-Chwen Lin, Shu-Ling Fu

**Affiliations:** 1 Institute of Traditional Medicine, National Yang Ming Chiao Tung University, Hsinchu 30010, Taiwan; jenny90680@gm.ym.edu.tw (L.-C.C.); yollywang@gmail.com (J.-Y.W.); owlherbs@yahoo.com.tw (C.-H.H.); 2 Department of Orthopaedics, Taipei Veterans General Hospital, Taipei 11217, Taiwan; 3 Department of Orthopaedics, Therapeutical and Research Center of Musculoskeletal Tumor, Taipei Veterans General Hospital, Taipei 11217, Taiwan; 4 Department of Life Sciences and Institute of Genome Sciences, National Yang Ming Chiao Tung University, Hsinchu 30010, Taiwan; chaohsiunglin@nycu.edu.tw; 5 National Research Institute of Chinese Medicine, Ministry of Health and Welfare, Taipei 11221, Taiwan

**Keywords:** anti-inflammation, *Chloranthus oldhamii* Solms, lipopolysaccharide, NF-κB, decandrin B

## Abstract

*Chloranthus oldhamii* Solms (CO) is a folk medicine for treating infection and arthritis pain but its pharmacological activity and bioactive compounds remain mostly uncharacterized. In this study, the anti-inflammatory compounds of *C. oldhamii* were identified using an LPS-stimulated, NF-κB-responsive RAW 264.7 macrophage reporter line. Three diterpenoid compounds, 3α-hydroxy-ent-abieta-8,11,13-triene (CO-9), 3α, 7β-dihydroxy-ent-abieta-8,11,13-triene (CO-10), and decandrin B (CO-15) were found to inhibit NF-κB activity at nontoxic concentrations. Moreover, CO-9 and CO-10 suppressed the expression of IL-6 and TNF-α in LPS-stimulated RAW 264.7 cells. The inhibitory effect of CO-9 on TNF-α and IL-6 expression was further demonstrated using LPS-treated bone marrow-derived macrophages. Furthermore, CO-9, CO-10, and CO-15 suppressed LPS-triggered COX-2 expression and downstream PGE2 production in RAW 264.7 cells. CO-9 and CO-10 also reduced LPS-triggered iNOS expression and nitrogen oxide production in RAW 264.7 cells. The anti-inflammatory mechanism of the most effective compound, CO-9, was further investigated. CO-9 attenuated LPS-induced NF-κB activation by reducing the phosphorylation of IKKα/β (Ser176/180), IκBα (Ser32), and p65 (Ser534). Conversely, CO-9 did not affect the LPS-induced activation of MAPK signaling pathways. In summary, this study revealed new anti-inflammatory diterpenoid compounds from C. *oldhamii* and demonstrated that the IKK-mediated NK-κB pathway is the major target of these compounds.

## 1. Introduction

Inflammation is a complex biological response of tissues to harmful stimuli, such as pathogens, damaged cells, or toxins. The function of inflammation is to remove the initial cause of cell injury and restore tissue functions and structures [1,2,3]. However, uncontrolled acute inflammation can turn into chronic inflammation leading to a variety of chronic inflammatory diseases, such as arthritis, neurodegenerative and atherosclerotic diseases [4,5]. Pattern recognition receptors (PRRs) (e.g., Toll-like receptors) are activated via interactions with specific molecular structures, including pathogen-associated molecular patterns (PAMPs) on pathogens. The stimulation of macrophages through TLR4 activation by lipopolysaccharides (LPS), a typical PAMP, leads to the expression of hallmark proinflammatory mediators, such as nitric oxide (NO), COX-2, PGE2, IL-6, and TNF-α, and triggers subsequent inflammatory events [6].

Several intracellular signal transduction pathways are known to mediate TLR4-dependent inflammation processes [7]; LPS-induced TLR4 activation triggers both MyD88-dependent and MyD88-independent signaling pathways. MyD88-dependent signaling cascades subsequently recruit tumor necrosis factor receptor-associated factor 6 (TRAF6) to activate transforming growth factor-activated kinase-1 (TAK1), which then phosphorylates and activates IκB kinase (IKK). Active IKKβ phosphorylates IκBα (inhibitor of NF-κB) and triggers the polyubiquitination and degradation of IκBα, leading to the exposure of the nuclear localization signals (NLS) of NF-κB proteins. Consequently, NF-κB is phosphorylated, translocates from the nucleus, and functions as a transcription regulator [7,8,9]. Conversely, TAK1 activation by LPS-mediated TLR4 signaling could also lead to the activation of mitogen-activated protein kinase (MAPK), which in turn activates activator protein 1 (AP-1) transcription factors [7,8]. Collectively, both NF-κB and AP-1 are crucial transcriptional activators of proinflammatory molecules involved in LPS-activated inflammatory processes [7].

Natural products have diverse pharmacophores and high complexity stereochemistry, rendering them crucial lead compounds in the development of new drugs. Many natural products identified from medicinal plants in folklore worldwide have been developed as therapeutic agents for clinical medication [10]. *Chloranthus oldhamii* Solms, belonging to the *Chloranthaceae* family, is distributed in forests at a low altitude of 500–1000 m and is a unique plant in Taiwan. It has been used as a folk medicine for treating infection, arthritis pain, and bone fractures. *C. oldhamii* is also used for external application to decrease blood stasis and pain in patients [2]. However, the pharmacological activity and bioactive compounds of *C. oldhamii* have rarely been studied. In this study, we identified the anti-inflammatory compounds of *C. oldhamii* using an established NF-κB activity reporter cell line. Furthermore, the anti-inflammatory effects and mechanisms of candidate compounds were investigated in LPS-treated macrophages.

## 2. Results

### 2.1. Isolation and Identification of Anti-Inflammatory Compounds from C. oldhamii

To identify anti-inflammatory ingredients from *C. oldhamii*, ethanol is considered as an ideal solvent for dissolving both polar and nonpolar compounds, which can extract most of the chemical components. Therefore, we first extracted *C. oldhamii* with ethanol, and then the ethanol extract was divided into four divisions according to the polarity by means of a liquid–liquid partition process. The effects of various fractions on NF-κB activation were examined using the LPS-responsive reporter cell line RAW 264.7/Luc-P1. According to the results, the ethanol, n-hexane, and ethyl acetate fractions of *C. oldhamii* all significantly suppressed NF-κB activation in LPS-stimulated RAW 264.7/Luc macrophages (Supplemental Figure S1). Due to a highly repetitive TLC profile, the n-hexane and ethyl acetate (EtOAc) fractions were combined and subsequently subjected to extensive chromatographic separations to obtain compounds CO-1 to CO-20. Based on the spectral data (including 1D-, 2D-NMR, and MS) and comparison with those reported in the literature, the isolated compounds were identified as myristicin (CO-1) [11], apiol (CO-2) [12], 4-allyl-2,6-dimethoxyphenyl glucopyranoside (CO-3) [13], methyl rosmarinate (CO-4) [14], rosmarinic acid (CO-5) [14], curzerenone (CO-6) [15], 1(10)Z,4Z-furanodiene-6-one (CO-7) [16], 3α-hydroxy-ent-abieta-7,13-diene (CO-8) [17], 3α-hydroxy-ent- abieta-8,11,13-trien-7-one (CO-11) [18], sessilifol J (CO-12) [19], 3α-hydroxy-ent-podocarp-8(14)-en-13-one (CO-13) [19], 3α-hydroxy-ent-podocarp-8(14)-en-7,13-dione (CO-14) [19], dolabradiene (CO-16) [20], isofraxidin (CO-17) [21], cinnamic acid (CO-18), caffeic acid (CO-19), and shizukaol C (CO-20) [22]. The structures of all identified compounds are shown in Supplemental Figure S2. The characterization of CO-9, CO-10, and CO-15 is further presented below.

CO-9, [α] -31.6 (c = 1, MeOH), has a molecular formula of C_20_H_30_O, as deduced by its EIMS and ^13^C NMR data. Its ^13^C NMR spectrum, including five methyl carbons, four secondary sp^3^ carbons, two tertiary sp^3^ carbons, one oxygenated tertiary sp^3^ carbon, two quaternary sp^3^ carbons, one secondary sp^2^ carbon, three tertiary sp^2^ carbons, and three quaternary sp^2^ carbons, indicated that CO-9 is a hydroxyl diterpene. Its ^1^H NMR spectrum showed signals of three tertiary methyls [δ 0.88, 1.05, and 1.17 (each s, H_3_-19, H_3_-18, and H_3_-20)]; an isopropyl group [δ 1.20 (d, J = 6.6 Hz, H_3_-16, 17), and 2.80 (m, H-15)]; an oxygenated methine proton [δ 3.28 (dd, J= 10.2/4.8 Hz, H-3)]; and a 1,2,4-trisubstituted aromatic ring [δ 6.88 (d, J = 1.2 Hz, H-1), 6.98 (dd, J = 7.8/1.2 Hz, H-12), and 7.14 (d, J = 7.8 Hz, H-11)]. The ^1^H and ^13^C NMR data of CO-9 are consistent with those of 3α-hydroxy-abieta-8,11,13-triene [23], but the specific rotations of CO-9 and 3α-hydroxy-abieta-8,11,13-triene ([α] + 37.3) [23] are opposite. Therefore, the structure of CO-9 was deduced as 3α-hydroxy-ent-abieta-8,11,13-triene.

CO-10 was determined to have a molecular formula of C_20_H_30_O_2_ based on the ^13^C NMR data and the [M]^+^ ion peak at *m*/*z* 302 in the EIMS, with one oxygen atom more than CO-9. Comparison of the ^1^H and ^13^C NMR spectra of CO-10 with those of CO-9 suggested that the main differences were from the B-ring attached to a hydroxy group at C-7 in CO-10, which was further verified by HMBC and NOESY experiments. The HMBC spectrum of CO-10 showed correlations of C-7 (δ 69.1) with H-5 (δ1.72) and H-14 (δ 7.16), confirming the location of the 7-hydroxy group. The NOESY spectrum showed correlations of H-7 (δ 4.75) with H3-20 (δ 1.12), indicating the configuration of 7α-hydroxy. Therefore, CO-10 was proposed as 3α, 7β-dihydroxy-ent-abieta-8,11,13-triene.

CO-15 was determined to have a molecular formula of C_20_H_32_O_3_ based on the ^13^C NMR data and the [M]^+^ ion peak at m/z 320 in the EIMS. The ^1^H NMR spectrum of CO-15 showed signals of three tertiary methyls [δ 0.83, 0.86, and 0.97 (each s, H_3_-20, H_3_-19, and H_3_-18)]; an isopropyl group [δ 0.83 (d, J = 7.2 Hz, H_3_-16), 17), 0.93 (d, J = 6.6 Hz, H_3_-17) and 1.77 (dd, J = 7.2/6.6 Hz, H-15)]; an oxygenated methine proton [δ 3.28 (dd, J = 10.2/4.8 Hz, H-3)]; and an olefinic proton (δ 6.72, s H-14). These signals are consistent with those of decandrin B [24]. The ^13^C NMR spectrum of CO-15 showed 20 signals, including characteristic signals of oxymethine carbon [δ 78.6 (d, C-3)], an oxygenated tertiary carbon [δ 71.8 (s, C-13)], and an α, β-unsaturated carbonyl group [δ 200.2 (s, C-7), 138.1 (s, C-8), and 139.9 (d, C-140.97)]. These signals are also consistent with those of decandrin B [24]. Therefore, the CO-15 structure was determined.

**Figure 1:**
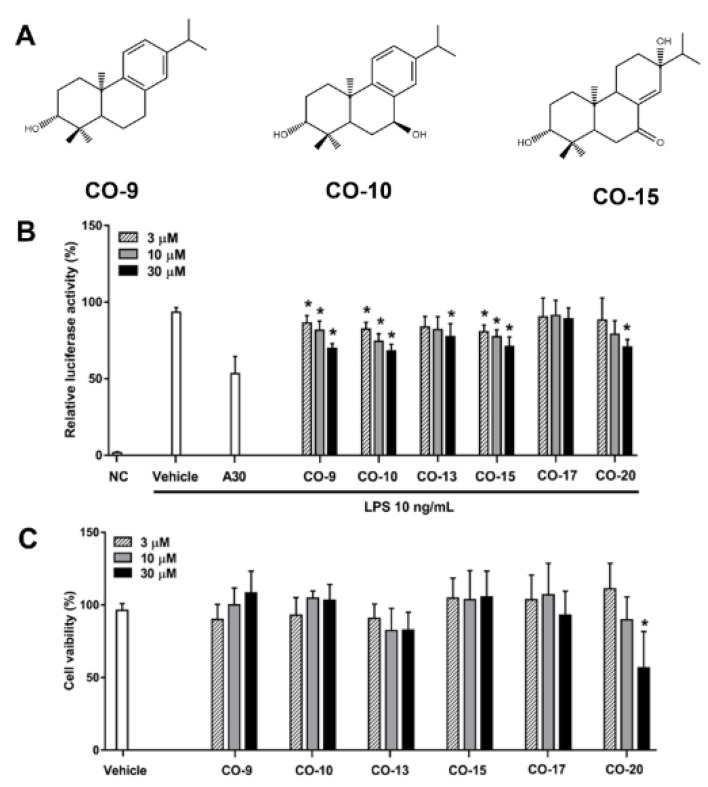
The effect of pure compounds from *C. oldhamii* on NF-κB activity in LPS-stimulated RAW264.7/Luc macrophages. (**A**) The chemical structures of CO-9, 10, and 15 identified from *C. oldhamii*. (**B**) RAW 264.7/Luc-P1 cells (3 × 10^5^ in MP-24 plates) were treated with various compounds or vehicle (0.1% DMSO) for 1 h before LPS treatment (10 ng/mL) for 6 h, then their luciferase activities were measured. Andrographolide at 30 μM (A30) was used as the positive control. (**C**) RAW264.7 macrophages (1 × 10^4^ cells in MP-96 plates) were treated with the indicated pure compounds of *C. oldhamii* or vehicle for 24 h, and then the cell viabilities of the treated cells were measured using MTT assays. Data are expressed as the mean ± SD from three independent experiments. * indicates significant differences versus the vehicle group (*p* < 0.05).

Except for compounds with low amounts (CO-8, 11~14, 16, 18~19) or previously reported active anti-inflammatory compounds (CO-4, 5) [14], all other compounds were subjected to anti-NF-κB activity evaluation. Among them, CO-9, CO-10, and CO-15 suppressed NF-κB activation in a concentration-dependent manner without causing cytotoxicity (Figure 1). CO-9, CO-10, and CO-15 are diterpenoids, and their anti-inflammatory activities have not been reported; thus, we further studied the anti-inflammatory activities and mechanisms of these diterpenoids.

### 2.2. Anti-Inflammatory Mechanism of Diterpenoid Compounds (CO-9, CO-10, and CO-15) in LPS-Induced Inflammatory Responses

**Figure 2:**
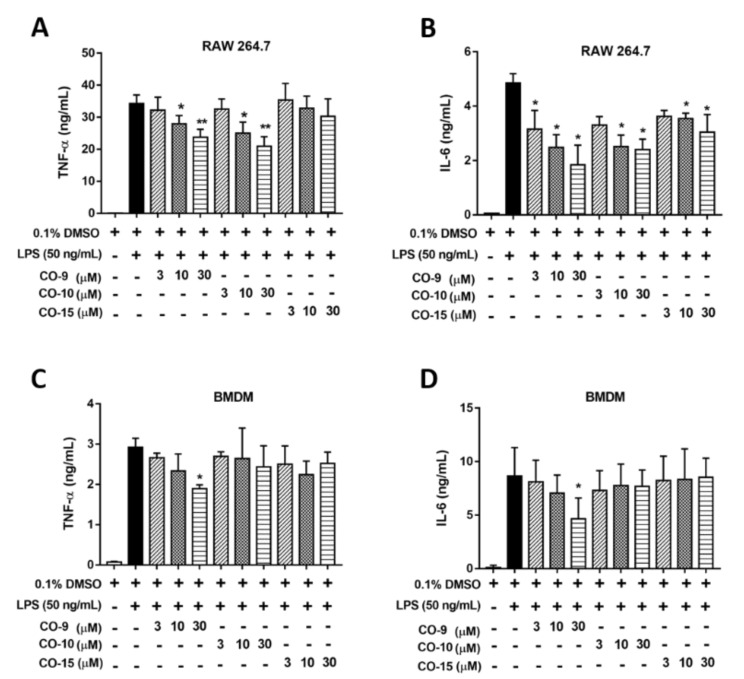
The effect of CO compounds on cytokine expression in LPS-stimulated RAW 264.7 macrophages and BMDMs. (**A**,**B**) RAW 264.7 macrophages (5 × 10^5^ in MP-6 plates) were treated with various concentrations of CO-9, CO-10, CO-15, or vehicle for 1 h before LPS treatment (50 ng/mL) for 24 h. The culture medium was assayed for the production of TNF-α (**A**) or IL-6 (**B**) using ELISA. (**C**,**D**) BMDMs (1 × 10^6^ cells/well in MP-6) were treated with various concentrations of CO-9, CO-10, CO-15, or vehicle for 1 h before LPS treatment (50 ng/mL) for 24 h. The expression of TNF-α (**C**) or IL-6 (**D**) in the culture medium was detected by ELISA. Data are expressed as the mean ± SD from three independent experiments. * (*p* < 0.05) and ** (*p* < 0.01) indicate significant differences versus the LPS plus vehicle group.

Because the NF-kB pathway is involved in the induction of hallmark proinflammatory cytokines such as TNF-α and IL-6, we investigated whether active compounds from *C. oldhamii* (CO-9, CO-10, CO-15) could suppress LPS-induced TNF-α and IL-6 expression. Our results showed that CO-9 and CO-10 suppressed LPS-induced production of TNF-α at both 10 μM and 30 μM (Figure 2A). CO-9 suppressed LPS-induced production of IL-6 in a concentration-dependent manner, while CO-10 and CO-15 suppressed LPS-induced production of IL-6 at 10 μM and 30 μM (Figure 2B). Bone marrow-derived macrophages (BMDMs) show more physiologically relevant characteristics than immortalized RAW 264.7 cells. Thus, we used BMDMs to validate whether CO-9, CO-10, and CO-15 could suppress the expression of proinflammatory cytokines. The results revealed that 30 μM CO-9 significantly suppressed the expression of TNF-α and IL-6 in LPS-stimulated BMDMs (Figure 2C,D).

**Figure 3:**
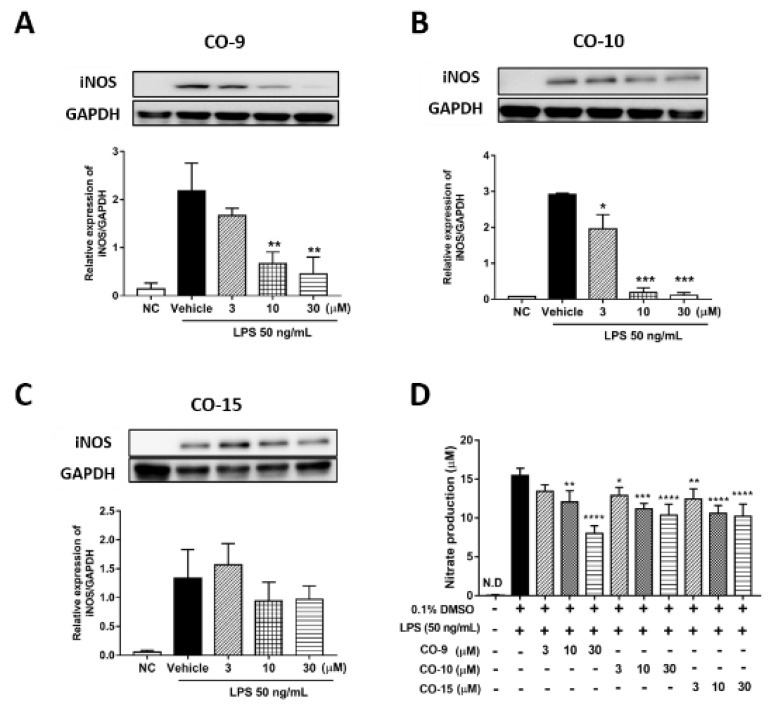
The effects of *C. oldhamii* compounds on iNOS expression and NO production in LPS-stimulated RAW 264.7 macrophages. (**A**–**C**) RAW 264.7 macrophages (5 × 10^5^ in MP-6 plates) were treated with various concentrations of CO-9, CO-10, CO-15, or vehicle for 1 h before LPS (50 ng/mL) treatment for 24 h. The iNOS expression levels were determined by Western blotting. (**D**) The culture supernatants of treated cells were analyzed for NO production using the Griess assay. Data are expressed as the mean ± SD from three independent experiments. * (*p* < 0.05), ** (*p* < 0.01), *** (*p* < 0.005), and **** (*p* < 0.001) indicate significant differences versus the LPS plus vehicle group.

The proinflammatory mediators nitric oxide (NO) and prostaglandin E2 (PGE2) are generated by inducible nitric oxide synthase (iNOS) and COX-2, respectively, in inflammatory processes. Both iNOS and COX-2 are downstream targets of NF-κB. Therefore, we investigated whether CO-9, CO-10, or CO-15 could suppress the production of iNOS, NO, COX-2, and PGE2. In unstimulated RAW 264.7 cells, the iNOS protein was undetectable. However, iNOS was obviously induced by LPS, and two diterpenoids (CO-9 and CO-10) significantly inhibited iNOS expression in a concentration-dependent manner (Figure 3A,C). As nitric oxide is easily oxidized to nitrate, we examined the effect of these diterpenoids on LPS-induced NO production by measuring the nitrate concentration in the culture medium. The results showed that CO-9, CO-10, and CO-15 significantly suppressed LPS-induced nitrate production in a concentration-dependent manner (Figure 3D).

**Figure 4:**
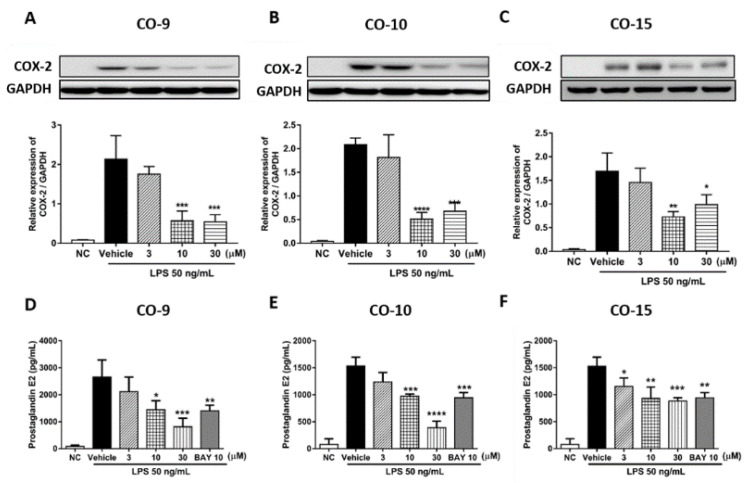
The effects of *C. oldhamii* compounds on COX-2 expression and PGE2 production in LPS-stimulated RAW 264.7 macrophages. (**A**–**C**) RAW 264.7 macrophages (5 × 10^5^ in MP-6 plates) were treated with various concentrations of CO-9, CO-10, CO-15, or vehicle for 1 h before LPS treatment (50 ng/mL) for 24 h. Afterwards, COX-2 expression levels were determined by Western blotting. (**D**–**F**) The culture supernatants of treated cells were analyzed for PGE2 levels using ELISA. BAY 11-7082 (10 μM) served as the positive control. Data are expressed as the mean ± SD from three independent experiments. * (*p* < 0.05), ** (*p* < 0.01), *** (*p* < 0.005), and **** (*p* < 0.001) indicate significant differences versus the LPS plus vehicle group.

Furthermore, COX-2 expression was also significantly reduced after treatment with these diterpenoids (Figure 4A–C). The downstream mediators of COX-2 and PGE2 were further measured by ELISA. Upon LPS treatment, PGE2 production was increased significantly in the culture medium. CO-9 and CO-10 treatments suppressed PGE2 expression at both 10 μM and 30 μM (Figure 4D,E), while CO-15 treatment suppressed PGE2 production at all tested concentrations (Figure 4F). In summary, these results suggest that diterpenoid compounds (CO-9, CO-10, and CO-15) extracted from *C. oldhamii* effectively inhibited LPS-induced proinflammatory COX-2 and iNOS pathways.

### 2.3. The Effect of the CO-9 Compound on the LPS-Activated MAPK Pathway in RAW 264.7 Macrophages

**Figure 5:**
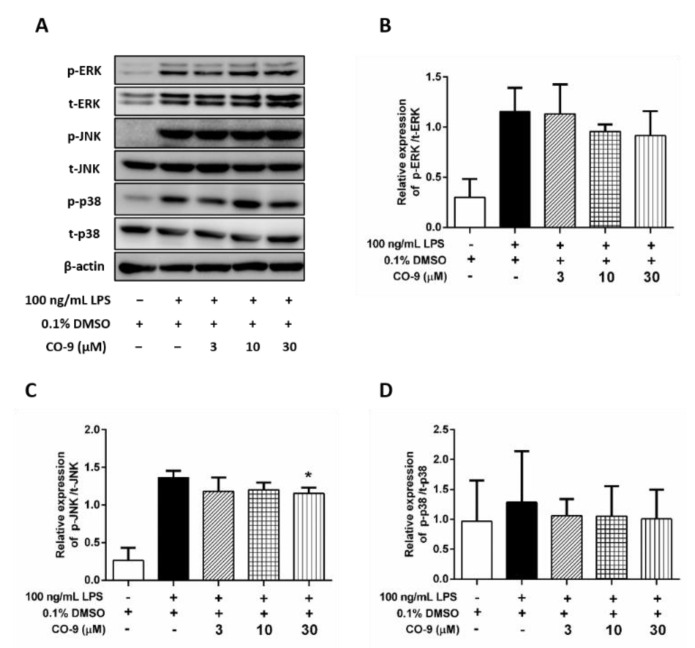
The effects of the CO-9 compound on MAPK signaling pathways in LPS-stimulated RAW 264.7 macrophages. RAW 264.7 cells (1 × 10^6^ cells in MP-6 plates) were treated with vehicle or CO-9 for 6 h, followed by LPS treatment (100 ng/mL) for 20 min. (**A**) Cell lysates were analyzed by Western blotting to detect the activation of ERK, JNK, and p38. Quantitative data on the expression of ERK (**B**), JNK (**C**), and p38 (**D**) are shown. Data are expressed as the mean ± SD from three independent experiments. * (*p* < 0.05) indicates significant differences versus the LPS plus vehicle group.

Based on the above data, CO-9 showed the strongest inhibition of LPS-induced inflammation among all tested CO compounds. We further examined the underlying mechanism of CO-9-mediated inflammatory responses in LPS-stimulated RAW 264.7 macrophages. The MAPK signaling pathways are known to play a key role in LPS-activated inflammatory responses. Hence, we also examined the effects of the CO-9 compound on the activation of the ERK1/2, JNK, and p38 signaling pathways. The expression levels of both active and total proteins were examined. As shown in Figure 5, CO-9 treatment did not affect the activation of MAPK signaling pathways in LPS-stimulated RAW 264.7 cells.

### 2.4. CO-9 Significantly Inhibits IKK-Mediated NF-κB Signaling Pathways in LPS-Stimulated RAW 264.7 Macrophages

**Figure 6:**
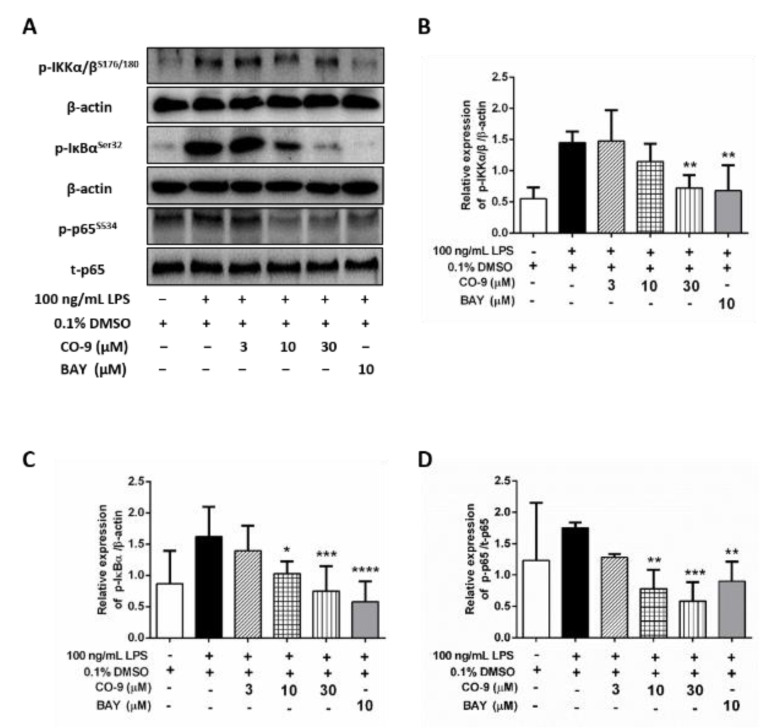
CO-9 significantly inhibits IKK-mediated NF-κB signaling pathways in LPS-stimulated RAW 264.7 macrophages. RAW 264.7 cells (1 × 10^6^ cells in MP-6 plates) were treated with vehicle or CO-9 for 6 h before incubation with LPS (100 ng/mL) for 20 min (p-IKKα/βSer176/180) or 30 min (p-IκBαSer32 and p-p65Ser534). (**A**) Cell lysates were analyzed by Western blotting to detect the phosphorylation of p-IKKα/βSer176/180, p-IκBαSer32, and p-p65Ser534. Quantitative data on the expression of p-IKKα/βSer176/180 (**B**), p-IκBαSer32 (**C**), and p-p65Ser534 (**D**) are shown. Data are expressed as the mean ± SD from three independent experiments. * (*p* < 0.05), ** (*p* < 0.01), *** (*p* < 0.005), and **** (*p* < 0.001) indicate significant differences versus the LPS plus vehicle group.

CO-9 efficiently suppressed NF-κB activity upon pretreatment, cotreatment, or posttreatment with LPS (Figure 1B; Supplemental Figure S3). The mechanism underlying CO-9 suppression of NF-κB activation was further examined. NF-κB is inactive when it is bound to IκB in the cytosol, but it becomes active after IκB is phosphorylated by IκB kinase (IKK) and subsequently degraded. Therefore, we determined the effect of the CO-9 compound on the activation of IKK and the phosphorylation of IκB after LPS treatment. As shown in Figure 6A–C, CO-9 reduced the LPS-activated phosphorylation of IKKα/β (Ser176/180) and IκBα (Ser32). It is known that phosphorylation of p65 at Ser534 leads to nuclear localization and transcriptional activation of NF-κB. Thus, we further examined the protein expression of phosphorylated and total p65 proteins. As shown in Figure 6A,D, CO-9 evidently suppressed the LPS-induced phosphorylation of p65 (Ser534) at both 10 and 30 μM in RAW 264.7 macrophages. Together, the above experiments confirmed that the CO-9 compound inhibited the activity of the NF-κB pathway by suppressing IKKα/β activation.

## 3. Discussion

*C. oldhamii* Solms is a folk medicine that is commonly used to treat infection, arthritis pain, and bone fractures. In this study, three diterpenoid compounds (CO-9, CO-10, and CO-15) isolated from *C. oldhamii* were demonstrated to suppress LPS-induced NF-κB activation at nontoxic concentrations (Figure 1). The anti-inflammatory potential of these compounds was further supported by their suppressive effects on the expression of TNF-α, IL-6, NO, iNOS, and COX-2 (Figure 2, Figure 3 and Figure 4). Notably, CO-9 was the most effective anti-inflammatory compound in this study because it concentration-dependently suppressed the expression of all proinflammatory mediators (TNF-α, IL-6, NO, iNOS, and COX-2) in LPS-stimulated RAW 264.7 cells (Figure 2, Figure 3 and Figure 4) and suppressed TNF-α and IL-6 expression in LPS-stimulated BMDMs (Figure 2C,D). Although a previous study described several compounds isolated from *C. oldhamii* that could suppress LPS-induced nitric oxide production [25], these compounds are structurally different from the diterpenoid compounds described in our study. Therefore, our study is the first to reveal anti-inflammatory diterpenoid compounds in *C. oldhamii*.

**Figure 7:**
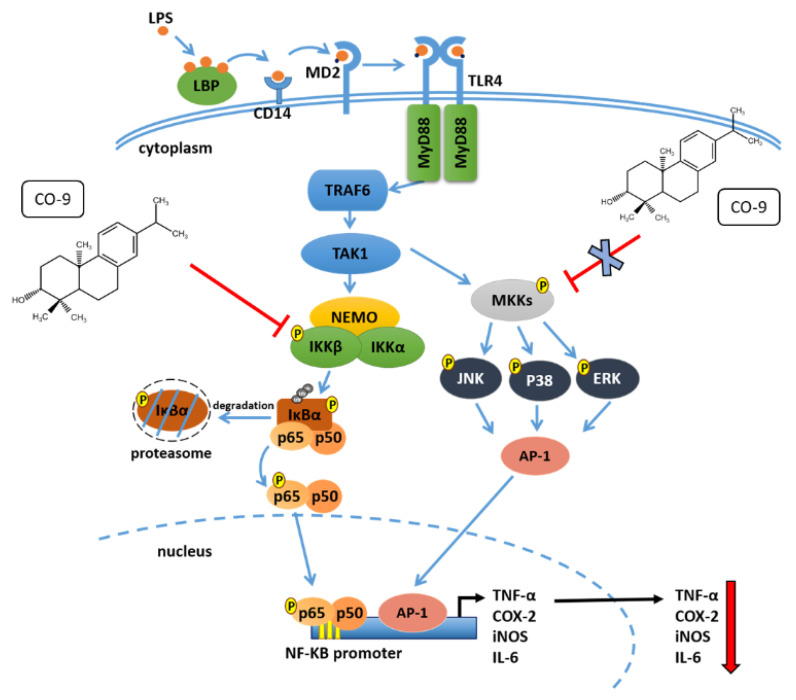
A proposed mechanism of the CO-9-mediated anti-inflammatory effect. In the LPS-induced TLR4 signaling pathway of macrophages, the CO-9 compound suppresses the activity of IKK, which reduces the phosphorylation of IκB(Ser32) and thus attenuates NF-κB-induced pro-inflammatory signaling pathways.

LPS-mediated TLR4 signaling could activate TAK1, which leads to the activation of both NF-κB- and MAPK-signaling pathways [7]. Our results showed that the CO-9 compound significantly suppressed the transcriptional activity of NF-κB and reduced the protein levels of p-IκB (Ser32) and p-p65 (Ser534) (Figure 1 and Figure 6), indicating that CO-9 suppressed NF-κB activation. In line with these observations, CO-9 downregulated several known NF-κB downstream proinflammatory mediators, iNOS, COX-2, TNF-α, and IL-6 (Figure 2, Figure 3 and Figure 4). Interestingly, the CO-9 compound exhibited no significant effect on the activation of the ERK-, p38-, and JNK-signaling pathways in LPS-stimulated RAW 264.7 cells (Figure 5). As CO-9 also suppressed the activation of IKKα/β, which is the upstream regulator of the NF-κB signaling pathway (Figure 6), our data suggest that CO-9 selectively targets TAK1-downstream IKKα/β-mediated NF-κB activation. Based on the above observation, a proposed mechanism of the CO-9-mediated anti-inflammatory effect is shown in Figure 7.

Based on current data, the CO-9 compound has stronger anti-inflammatory activity than the CO-10 and CO-15 compounds. CO-9, CO10, and CO-15 are diterpenoids that consist of four isoprenes. The structures of CO-9 and CO-10 are very similar; the only difference between them is that carbon number seven of CO-10 has a hydroxyl group. However, the structure of CO-15 is more distinct from both the CO-9 and CO-10 compounds. The activity of drugs is highly related to their chemical structures, which determines their binding capacity to target proteins. We speculate that the additional hydroxyl group at carbon number seven in CO-10, compared with CO-9, may hinder the target-binding ability of CO-10 and thus decrease its anti-inflammatory activity.

Our study reveals several novel anti-inflammatory diterpenoid compounds in *C. oldhamii*. A previous study described the identification of twelve ent-Abietane diterpenoids isolated from *C. oldhamii* [25], but the structures of these compounds are different from those in our study. Furthermore, the paper mainly focuses on the structural features of these compounds, and their anti-inflammatory activity was mainly determined by the inhibition on LPS-induced nitric oxide production [25]. In addition, three new phloroglucinol-diterpene adducts, chlorabietols A−C, have been isolated from the roots of *C. oldhamii*, and they were shown to inhibit protein tyrosine phosphatase 1B (PTP1B) in vitro [26]. According to the literature, other species of Chloranthus also contain anti-inflammatory components. The sesquiterpene compounds isolated from *Chloranthus japonicus* and the water extracts of *Chloranthus serratus* have been shown to suppress inflammatory responses by inhibiting the NF-κB signaling pathway [4,27]. Together, current data indicate that Chloranthus represents a rich resource of anti-inflammatory natural compounds.

In summary, our study discovers new anti-inflammatory diterpenoid compounds from *C. oldhamii* Solms, demonstrating the anti-inflammatory potential of this folk medicine. Among them, the CO-9 compound is the most effective and selectively targets the IKK-mediated NK-κB pathway, representing a promising anti-inflammatory lead compound. To fully explore the potential application of CO-9 as an anti-inflammatory agent, the pharmacokinetics, toxicity profiles, and in vivo anti-inflammatory activity of CO-9 certainly merit further investigation.

## 4. Materials and Methods

### 4.1. Chemicals and Antibodies

Ultrapure lipopolysaccharide (LPS) from E. coli O111:B4 was purchased from InvivoGen (San Diego, CA, USA). Dimethyl sulfoxide (DMSO), MTT [3-(4,5-dimethylthiazol-2-yl)-2,5-diphenyltetrazolium bromide], protease inhibitors, albumin bovine serum (BSA), and Bradford reagents were purchased from Sigma-Aldrich (Saint Louis, MO, USA). Anti-glyceraldehyde-3-phosphate dehydrogenase (GAPDH) antibody (MAB374) was obtained from Sigma-Aldrich. Anti-COX-2 (#12282), anti-phospho-IκB (Ser32) (#2859), anti-phospho-NF-κB p65 (Ser534) (#3033), anti-phospho-ERK (Thr202/Tyr204) (#9101), anti-ERK (#9102), anti-phospho-JNK (Thr183/Tyr185) (#9251), anti-JNK (#9252), anti-phospho-p38 (Thr180/Tyr182) (#9211), anti-p38 (#9212), and anti-β actin antibodies were purchased from Cell Signaling Inc. (Danvers, MA, USA). Anti-iNOS (ab15323) and anti-NF-κB p65 antibodies (ab7970) were purchased from Abcam (Cambridge, UK).

### 4.2. Extraction and Isolation of Compounds from C. oldhamii Solms

Whole plants of *C. oldhami* Solms were collected from Hsinchu County, Taiwan, in April 2016 and verified by comparison of a voucher specimen (TAIE-17362) of C. *oldhami* Solms deposited in the Herbarium of Endemic Species Research Institute, Taiwan [28]. The air-dried whole plant of *C. oldhamii* (1.4 kg) was cut into small pieces of about 5 cm and extracted with EtOH (40 L) three times (for 16 h each time) at 50 °C. The solutions were then combined and the solvents were removed to obtain EtOH extract. Then, the EtOH extract (270 g) was suspended in H_2_O (1 L) and extracted successively with n-hexane, ethyl acetate, and n-butanol (each 1 L × 3) to obtain the n-hexane, EtOAc, n-BuOH, and H_2_O fractions. The extraction procedures were adopted from the literature [14]. The n-hexane and EtOAc fractions showed a highly repetitive profile in the TLC analysis; therefore, we combined them together for the subsequent chromatography separation experiments to isolate pure compounds as described in “Supplementary Methods”.

### 4.3. Cell Culture

The RAW 264.7 murine macrophage cell line purchased from Bioresource Collection and Research Center (Hsinchu, Taiwan) was cultured at 37 °C in 5% CO_2_ in Dulbecco’s Modified Eagle’s Medium (DMEM; Gibco, Grand Island, NY, USA) containing 10% heat-inactivated fetal bovine serum (FBS; Gibco, Grand Island, NY, USA), 100 units/mL penicillin, 100 µg/mL streptomycin, 2 mM L-glutamine, and 1 mM sodium pyruvate (Gibco). RAW 264.7/Luc-P1 cells were established and cultured as described previously [29].

### 4.4. Luciferase Reporter Assay

Vehicle- or drug-treated RAW 264.7/Luc-P1 cells were lysed in passive lysis buffer (Promega, Wisconsin, USA) and analyzed using luciferase assays as described previously [29]. The data are expressed as relative activities versus the LPS plus vehicle group.

### 4.5. Cell Viability Assay

Cell viability was measured using MTT assays as described previously [14]. The data are expressed as relative viability versus the untreated group.

### 4.6. Preparation of Bone Marrow-Derived Macrophages (BMDMs)

Bone marrow-derived macrophages were isolated from the femur and tibia bones of 6- to 8-week-old C57BL/6 male mice using a previously described protocol [30]. BMDMs were grown in RPMI 1640 medium with 10% heat-inactivated FBS, 1% nonessential amino acids (NEAAs), 100 units/mL penicillin, 100 μg/mL streptomycin, 2 mM L-glutamine, and 20 ng/mL macrophage colony-stimulating factor (R&D Systems). The animal study was approved by the Institutional Animal Care and Use Committee (IACUC, NO. 1051219) at National Yang-Ming University (Taipei, Taiwan).

### 4.7. Enzyme-Linked Immunosorbent Assay (ELISA)

The culture supernatants of treated cells were collected, and the level of TNF-α or IL-6 was measured using commercial ELISA kits purchased from R&D Systems and BD (Franklin Lakes, NJ, USA), respectively. The A450 nm and A540 nm (reference absorbance) were measured using a Model 680 microplate reader (TECAN, Männedorf, Zürich, Switzerland).

### 4.8. Western Blotting

The treated cells were lysed in RIPA buffer containing protease inhibitors and then quantitated using Bradford assays. Equivalent amounts of cell lysate (50 μg) from each sample were analyzed by 10% SDS-PAGE, transferred to PVDF membranes, and reacted with the appropriate primary antibody and HRP-conjugated secondary antibody using the protocol described previously [14]. The signals were detected by enhanced chemiluminescence (ECL) reagent (GE Healthcare, Chicago, IL, USA). Data were quantified using the ImageJ program, version 1.52 (NIH, Bethesda, Rockville, MD, USA).

### 4.9. Statistical Analysis

All data are expressed as the mean ± SD of at least three independent experiments. Statistical analysis of various concentrations at the same time was performed using one-way analysis of variance (ANOVA) followed by Dunnett’s correction performed post hoc. The *p* values of < 0.05 represent a statistically significant difference.

## Supplementary Materials

The following are available online: Supplementary methods; Figure S1: The fraction procedure for *Chloranthus oldhamii* and the effects of different fractions on NF-κB activity in LPS-stimulated macrophages; Figure S2: The structures of isolated compounds from *C. oldhami*; Figure S3: The effect of CO-9 on NF-κB activity in LPS-stimulated macrophages under different treatments.

## Sample Availability

Samples of the compounds are available from the authors.
