# Supplementary material for: Diterpenoid Compounds Isolated from Chloranthus oldhamii Solms Exert Anti-Inflammatory Effects by Inhibiting the IKK/NF-κB Pathway
Source: Molecules. 2021 Oct 29;26(21):6540. doi: 10.3390/molecules26216540 (PMC8588554; doi:10.3390/molecules26216540)
Supplement: Supplementary file 1 [file molecules-26-06540-s001.zip › molecules-1440400-supplementary.pdf]

molecules

MDPI

Article

Diterpenoid compounds isolated from *Chloranthus oldhamii* Solms exert anti-inflammatory effects by inhibiting the IKK/NF-κB pathway

Lin-Chieh Chiu [1], Jir-You Wang, [1,2,3] Chao-Hsiung Lin [4], Chung-Hua Hsu [1], Lie-Chwen Lin [5,*], and Shu-Ling Fu [1,*]

[1] Institute of Traditional Medicine, National Yang Ming Chiao Tung University, Hsinchu 30010, Taiwan; jenny90680@gm.ym.edu.tw (L.-C.C.); yollywang@gmail.com (J.-Y.W.); owlherbs@yahoo.com.tw (C.-H.H.)
[2] Department of Orthopaedics, Taipei Veterans General Hospital, Taipei 11217, Taiwan
[3] Department of Orthopaedics, Therapeutical and Research Center of Musculoskeletal Tumor, Taipei Veterans General Hospital, Taipei 11217, Taiwan
[4] Department of Life Sciences and Institute of Genome Sciences, National Yang Ming Chiao Tung University, Hsinchu 30010, Taiwan; chaohsiunglin@nycu.edu.tw
[5] National Research Institute of Chinese Medicine, Ministry of Health and Welfare, Taipei 11221, Taiwan
* Correspondence: lclin@nricm.edu.tw (L.-C.L.); slfu@nycu.edu.tw (S.-L.F.),
Tel.: +886-2-2820-1999 (ext. 7101) (L.-C.L.); +886-2-28267177 (S.-L.F.)

Citation: Chiu, L.-C.; Wong, J.-Y; Lin, C.-H.; Hsu, C.-H.; Lin, L.-C.; Fu, S.-L. Diterpenoid compounds isolated from *Chloranthus oldhamii* exert anti- inflammatory effects by inhibiting the IKK/NF-κB pathway. *Molecules* **2021**, *26*, 6540. https://doi.org/10.3390/molecules26216540

Academic Editor: Maria da Graça Costa G. Miguel

Received: 18 October 2021
Accepted: 28 October 2021
Published: 29 October 2021

Publisher's Note: MDPI stays neutral with regard to jurisdictional claims in published maps and institutional affiliations.

Copyright: © 2021 by the authors. Submitted for possible open access publication under the terms and conditions of the Creative Commons Attribution (CC BY) license (https://creativecommons.org/licenses/by/4.0/).

Supplementary information: Methods

Isolation of compounds from *Chloranthus oldhamii* Solms

The *n*-hexane and EtOAc fractions showed a highly repetitive profile in the TLC analysis; thus, we combined them together (as fr. HE) for the following chromatography separation experiments. Fr. HE (105 g) was then subjected to column chromatography on silica gel (8 × 65 cm) and eluted with a solvent mixture of *EtOAc*/*n*-hexane (in ratios of 10, 20, 30, 40, 65, 80, and 100%, each 3.5 liter) and then with 5%, 10%, and 20% MeOH/EtOAc to supply 12 fractions (Frs. 1–12). Fr. 4 (18 g) was chromatographed on a silica gel (eluted with 20%–80% EtOAc/*n*-hex) to supply frs. 4-1~4-8. Fr. 4–3 was chromatographed on a semipreparative HPLC (*LiChrospher® RP-select B*, 10 × 250 mm, 5 µm, eluent: 80% ACN/H_2O, flow rate: 4.0 ml/min) to give CO-8 (3.9 mg) and CO-9 (21.2 mg). Fr. 4–5 and Fr. 4–6 were individually chromatographed on a semipreparative HPLC (*LiChrospher® RP-select B*, 10 × 250 mm, 5 µm, eluent: 45% ACN/H_2O, flow rate: 3.7 ml/min). Afterwards, CO-11 (2.8 mg) was isolated from Fr. 4–5 and CO-10 (61.0 mg) and CO-12 (4.7 mg) were isolated from Fr. 4–6. 4-9 was chromatographed on a Sephadex-LH-20 (eluted with acetone) and preparative TLC (developed with 60% EtOAc/*n*-hex) to give CO-15 (46.0 mg). Fr. 9 was chromatographed on a silica gel (eluted with 2%-10% MeOH/$CHCl_3$) to give Frs. 9–1~9-6. Fr. 9–4 was chromatographed on a Sephadex-LH-20 (eluted with acetone) and on a semipreparative HPLC (Cosmosil 5C18-AR-II, 10 × 250 mm, 5 µm, solvent: 35~40% ACN/0.1% formic acid for 20 min, flow rate: 3.7 ml/min) to supply CO-13 (3.6 mg), CO-14 (2.3 mg), and CO-17 (9.6 mg). Fr. 9–5 was chromatographed on a Sephadex-LH-20 (eluted with 50% EtOAc/MeOH) and on a semipreparative HPLC (Cosmosil 5C18-AR-II, 10 × 250 mm, 5 µm, eluent: 50% ACN/0.1% formic acid, flow rate: 3.7 ml/min) to supply CO-18 (2.8 mg) and CO-20 (32.5 mg). Using a similar chromatographic technique, CO-1 (7.5 mg), CO-2 (74.3 mg), CO-6 (6.8 mg), CO-7 (7.2 mg), and CO-16 (1.5 mg) were isolated from Fr. 3; CO-19 (3.5 mg) was isolated from Fr. 10; and CO-3 (29.8 mg), CO-4 (13.8 mg), and CO-5 (48.5 mg) were isolated from Fr. 12.

Supplementary Information: Figures

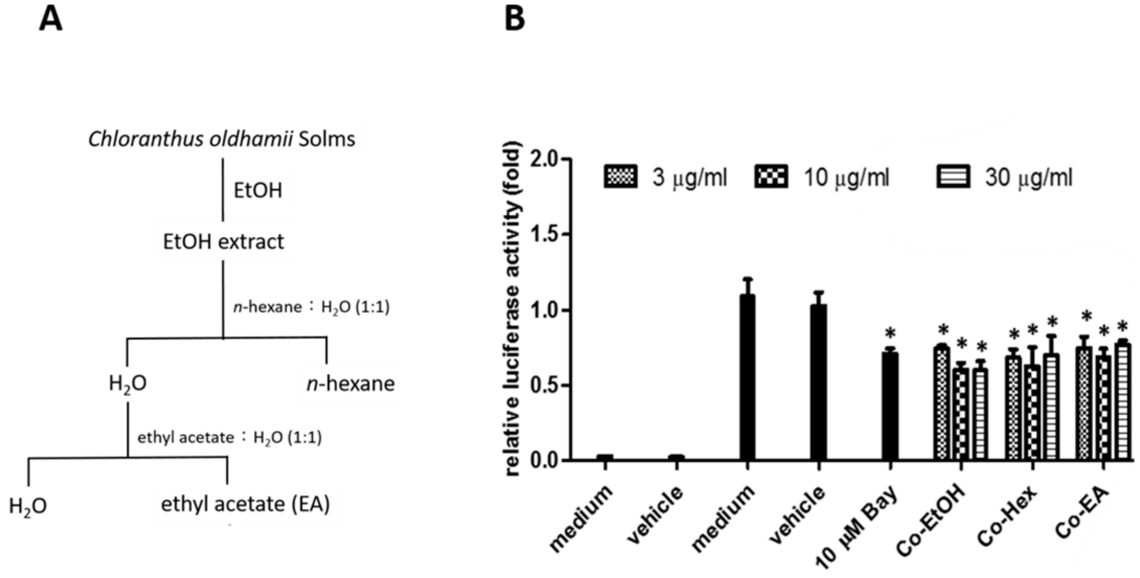

Supplementary Figure S1. The fraction procedure for *Chloranthus oldhamii* and the effects of different fractions on NF-κB activity in LPS-stimulated macrophages. (A) The outline of the fractionation scheme. (B) RAW264.7/Luc-P1 cells ($3x10^5$ in MP-24 plates) were treated with different herbal fractions or vehicle (0.1% DMSO) for 1 h, followed by LPS treatment (10 ng/ml) for 6 h. Bay117082 (10 μM) served as the positive control. Data are expressed as the mean ± SD from three independent experiments. * indicates a significant difference versus the vehicle group ($p < 0.05$).

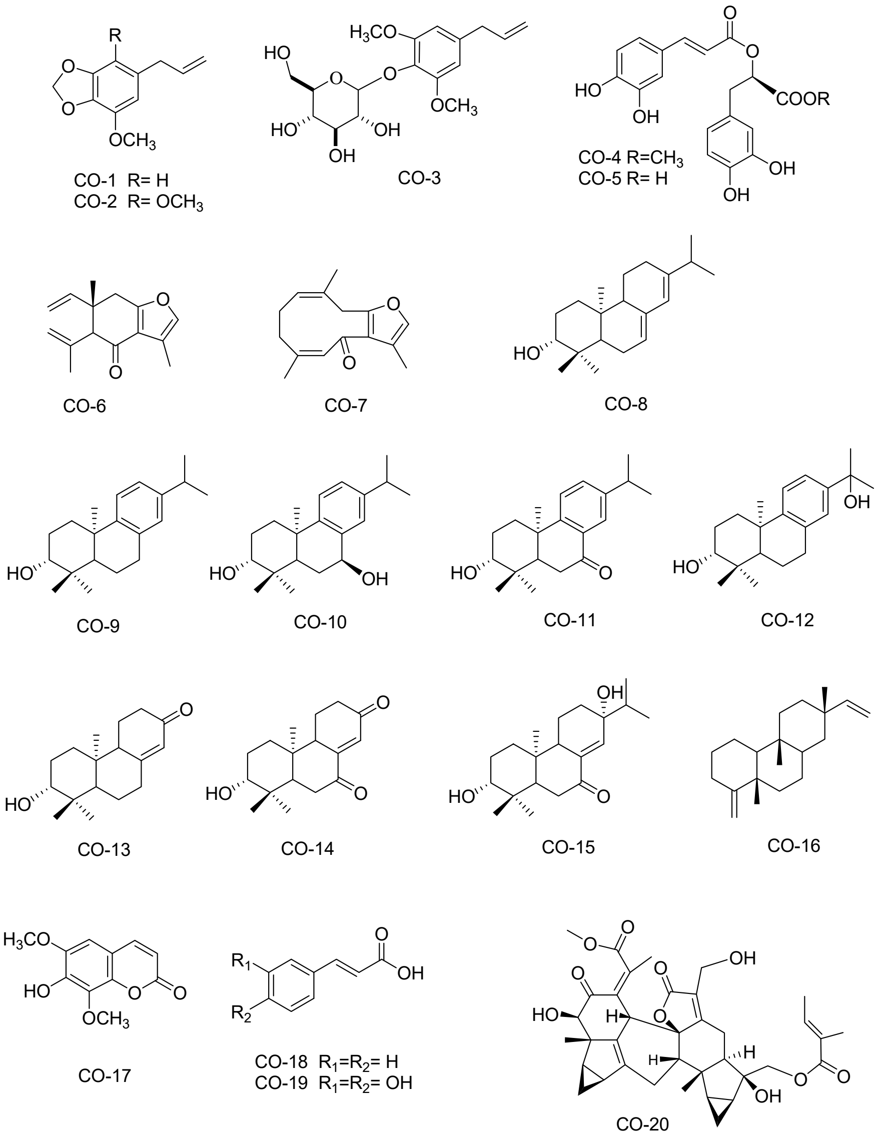

Supplementary Figure S2. The structures of isolated compounds from *C. oldhamii*. CO-1: myristicin, CO-2: apiol, CO-3: 4-allyl-2,6-dimethoxyphenyl glucopyranoside, CO-4: methyl rosmarinate, CO-5: rosmarinic acid, CO-6: curzerenone, CO-7: 1(10)Z,4Z-furanodiene-6-one, CO-8: 3α-hydroxy-ent-abieta-7,13-diene, CO-9: 3α-hydroxy-ent-abieta-8,11,13-triene, CO-10: 3α, 7β-dihydroxy-ent-abieta-8,11,13-triene, CO-11: 3α-hydroxy-ent- abieta-8,11,13-trien-7-one, CO-12: sessilifol J, CO-13: 3α-hydroxy-ent-podocarp-8(14)-en-13-one, CO-14: 3α-hydroxy-ent-podocarp-8(14)-en-7,13-dione, CO-15: decandrin B, CO-16: dolabradiene, CO-17: isofraxidin, CO-18: cinnamic acid, CO-19: caffeic acid, and CO-20: shizukaol C.

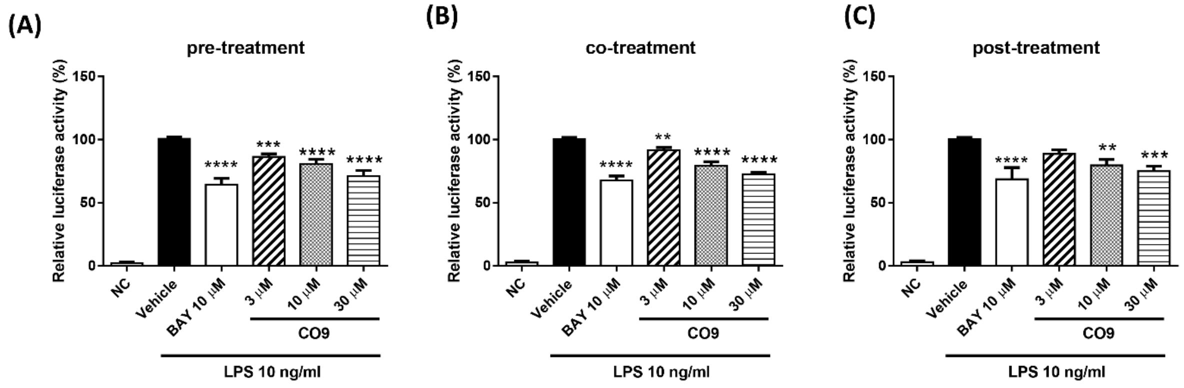

Supplementary Figure S3. The effect of CO-9 on NF-κB activity in LPS-stimulated macrophages under different treatments. (A) RAW264.7 cells ($3x10^5$ in MP-24 plates) were treated with various concentrations of drug or vehicle (0.1% DMSO) for 1 h before LPS treatment (10 ng/ml) for 6 hours. (B) Cells were simultaneously treated with 10 ng/ml LPS and various concentrations of drug or vehicle for 6 h. (C) Cells were stimulated with 10 ng/ml LPS for 1 h, followed by treatment with various concentrations of the indicated compounds or vehicle for another 6 h. Data are expressed as the mean ± SD from three independent experiments. (*) $p < 0.05$, (**) $p < 0.01$, (***) $p < 0.005$, and (****) $p < 0.0001$ indicate significant differences versus the LPS plus vehicle group.
